# Examining changes in maternal and child health inequalities in Ethiopia

**DOI:** 10.1186/s12939-017-0648-1

**Published:** 2017-08-22

**Authors:** Alemayehu A. Ambel, Colin Andrews, Anne M. Bakilana, Elizabeth M. Foster, Qaiser Khan, Huihui Wang

**Affiliations:** 1 The World Bank, 1818 H Street, Washington, DC 20046 USA; 2 The World Bank, Addis Ababa, Ethiopia

**Keywords:** Maternal and child health, Health inequalities, Health care utilization

## Abstract

**Background:**

Ethiopia has made considerable progress in maternal, newborn, and child health in terms of health outcomes and health services coverage. This study examined how different groups have fared in the process. It also looked at possible factors behind the inequalities.

**Methods:**

The study examined 11 maternal and child health outcomes and services: stunting, underweight, wasting, neonatal mortality, infant mortality, under-5 mortality, measles vaccination, full immunization, modern contraceptive use by currently married women, antenatal care visits, and skilled birth attendance. It explored trends in inequalities by household wealth status based on Demographic and Health Surveys conducted in 2000, 2005, 2011, and 2014. The study also investigated the dynamics of inequality, using concentration curves for different years. Decomposition analysis was used to identify the role of proximate determinants.

**Results:**

The study found substantial improvements in health outcomes and health services: Although there is still a considerable gap between the rich and the poor, inequalities in health services have been reduced. However, child nutrition outcomes have mainly improved for the rich. The changes observed in wealth-related inequality tend to reflect the changing direct effect of household wealth on child health and health service use.

**Conclusions:**

The country’s efforts to improve access to health services have shown some positive results, but attention should now turn to service quality and to identifying multisectoral interventions that can change outcomes for the poorest.

**Electronic supplementary material:**

The online version of this article (doi:10.1186/s12939-017-0648-1) contains supplementary material, which is available to authorized users.

## Background

Improving maternal and child health was integral to the Millennium Development Goals (MDGs) for 1990–2015: Goal 4 called for a two-thirds reduction in under-5 mortality, and Goal 5 for a 75% reduction in maternal mortality. The goals received global attention as countries and their international development partners mobilized support to, e.g., expand childhood immunization and increase the availability and utilization of maternal health services.

Ethiopia made considerable progress towards achieving the targets. In 2011 the Center for Global Development reported satisfactory progress on all goals and ranked Ethiopia 33rd of 137 countries, with an MDG progress index of 4.5 on a scale of zero to 8 points [1]. According to a 2012 UN report, all the MDG targets in Ethiopia were either on track or likely to be on track [2].

The 2014 mini-Demographic and Health Survey (DHS) found reductions in child undernutrition and child mortality and increased coverage of maternal, newborn, and child health (MNCH) services like antenatal care, contraceptive prevalence, and skilled birth attendance (SBA) [3]. With the MDG period behind us and new targets set in the Sustainable Development Goals (SDGs), attention is now turning to whether the achievements recorded were inclusive [4]. Of the few studies on health inequalities in Ethiopia, most analyzed just one or two indicators at the national level [5–8], while others examined one or two indicators for a specific region or city [6, 9].

In this study, we provide evidence of the dynamics of MNCH inequalities. The study contributes to the empirical evidence by adding a more detailed inequality analysis using data from a series of comparable recent surveys. The surveys allow us to analyze the changes in inequalities over a period that overlaps with most of the MDG period. We also examine the contribution of socioeconomic determinants of maternal and child health outcomes.

## Methods

### Indicators and definitions

Table 1 presents the six health status and five health service indicators analyzed in this study, chosen based on their relevance to the health MDGs and on the availability of data. For health status, there are three child undernutrition and three mortality indicators: stunting, wasting, underweight, neonatal mortality rate (NMR), infant mortality rate (IMR), and under-5 mortality rate (U5MR). The service indicators cover child immunization and maternal health services; they are measles vaccination, full immunization, prevalence of modern contraceptive use by married women, four or more antenatal care visits from a skilled professional (ANC4+), and delivery assistance from a skilled birth attendant (SBA). Additional file 1: Table 1a and b present details of the indicators and terms as used in the analysis.

**Table 1:** Maternal and child health indicators analyzed in this study

Indicators	Definition
Stunting	Percentage of children with a height-for-age z-score < −2 standard deviations from the reference median
Wasting	Percentage of children with a weight-for-height z-score < −2 standard deviations from the reference median
Underweight	Percentage of children with a weight-for-age z-score < −2 standard deviations from the reference median
Neonatal mortality rate (NMR)	The number of neonates dying before reaching 28 days of age per 1000 live births
Infant mortality rate (IMR)	The number of deaths among children under 12 months of age per 1000 live births
Under-5 mortality rate (U5MR)	The number of deaths among children under 5 years of age per 1000 live births
Measles vaccination	Percentage of children aged 12 to 23 months who received measles^a^,^b^
Full immunization	Percentage of children aged 12 to 23 months who received BCG, measles, and three doses of polio and DPT^b^,^a^
Contraceptive prevalence (modern method)	Percentage of currently married women aged 15 to 49 who currently use a modern method of contraception
Antenatal care visits, 4 or more (ANC4+)	Percentage of mothers aged 15 to 49 who had a live birth in the past 5 years who received at least 4 antenatal care visits from any skilled personnel during pregnancy for the most recent birth
Skilled birth attendant (SBA)	Percentage of live births to mothers aged 15 to 49 in the past five years that were attended by skilled health attendant

*Notes*:^a^Immunizations are either verified by card or based on recall of respondent. ^b^ Data not collected in 2014 mini-DHS survey

### Data sources and variable construction

The data are from four Ethiopia Demographic and Health Surveys (DHSs), in 2000, 2005, 2011, and 2014. These population-based surveys target mainly women of child-bearing age (15 to 49) but also collect some data about the household and some from men in the same age range. The main survey concerns are fertility, family planning, infant and child mortality, maternal and child health, and nutrition. Since 2000, they have been conducted about every 5 years. The first three surveys each sampled about 15,000 women from about the same number of households. The sample for the 2014 survey was about half that of the previous rounds. Much of the data used in this study comes from the women’s questionnaire, which compiles a comprehensive birth history for each woman, from antenatal care and delivery attendance through child survival and vaccination. The questionnaires used in the different surveys are standard and comparable. The data provide nationally representative information on the variables we selected for this study.

The primary qualifying variable is wealth ranking. We looked at socioeconomic inequalities in health by wealth ranking between the worse-off (bottom 40%) and the better-off (top 60%) and between the poorest (1st quintile) and the richest (5th quintile). These are computed from the household wealth index available with the data [10].

To evaluate child undernutrition, we computed anthropometric indicators based on the WHO 2006 growth standards: We calculated height-for-age, height-for-weight, and weight-for-age z-scores and then stunting, wasting, and underweight levels for children aged 0 to 59 months. Child mortality rates (IMR, NMR, and U5MR) are calculated using the standard DHS methodology, using data on all child deaths in the 5-year period preceding each survey [8]. We calculated the prevalence of modern contraception use by currently married women, antenatal care (most recent birth), and skilled birth attendance (all births in last 5 years) from the questionnaire administered to all women aged 15–49 years in the household.

### Data analysis

For a more complete picture, we considered a combination of the approaches often used in inequality studies because each approach has some limitations that can lead to different conclusions [11, 12]. Our analysis started with how absolute and relative inequalities between the poor and the rich have widened or narrowed over time. Then, we looked at the concentration curve and concentration index, which capture inequality across a continuous spectrum of wealth, and what they reveal about the changing pattern of inequality over time. Finally, we looked at the decomposition of the concentration indexes to see the changing role of various demographic and socio-economic factors in the observed wealth-based inequality in health services and outcomes.

We computed absolute inequalities from rate differences between the poor and the rich, defined both as the bottom 40% versus the top 60% and as the poorest quintile versus the richest.

The difference-in-differences comparison is as follows: Let I_xt_ be the value of the indicator for group x (either r = rich or p = poor) in time period t (either *t* = 0 first survey or t = T latest survey). We perform an F-test of the hypothesis:

1 $$ {I}_{p0}-{I}_{r0}={I}_{pT}-{I}_{rT} $$

Second, instead of the difference between the value of the indicator for rich and poor, the ratio of the values was used. This emphasizes the difference between indicators where both groups have very low values. The hypothesis tested was:

2 $$ \frac{I_{p0}}{I_{r0}}=\frac{I_{pT}}{I_{rT}} $$

The analysis of inequality based on absolute and relative gaps was limited to binary distinctions: rich vs. poor. It was thus somewhat sensitive to the definition of the binary distinction and also did not allow for analysis of inequality across the whole range of wealth outcomes. For example, the comparison of the bottom quintile to top quintile entirely ignored any changes in the health indicator for the middle three-fifths of the population. If we see a decrease in inequality between the bottom 40% and the top 60%, we do not know whether this was due to improvements for the poorest of the poor or for those closer to the middle of the income distribution. Therefore, we used concentration curves to illustrate the movement of wealth inequalities in health across the entire range of wealth between the earliest and latest surveys. We also used concentration indices (C), which quantify the degree of inequality in this analysis, and observed how they changed between the earliest and latest surveys [13].

A concentration curve plots the inequality of an outcome variable against another factor, here household wealth. It is constructed like a Lorenz curve, which illustrates the degree of inequality in a certain variable such as income. The population is sorted according to wealth, and the cumulative percentage of the indicator (y-axis) is plotted against the cumulative percentage of the population (x-axis). Thus if the bottom 5% of children by household wealth account for only 1% of the measles vaccinations, the first point on the curve is (0.05, 0.01). *Continuing*: if the bottom 10% of children (cumulatively) account for 3% of measles vaccinations, the second point is (0.1, 0.03), and construction of the curve continues in the same way. The concentration curve is often plotted against the 45-degree line, the line of equality the concentration curve would follow if health outcomes were evenly distributed across the wealth rankings.

We would expect the concentration curve for positive health indicators (immunization, maternal health services) to lie below the line of equity (poorer households account for a disproportionately low number of fully vaccinated children or attended births). This is shown in the measles examples above, where both points plotted lie below the 45-degree line. Conversely, we would expect the concentration curve for a negative health indicator (child mortality, malnutrition) to lie above the line of equality (poorer households account for a disproportionately high number of child deaths). We expect that the bottom 5% of children, ranked by wealth, account for more than 5% of underweight children and thus the point is above the 45-degree line. A concentration curve that moves closer to the line of equality over time indicates decreasing inequality.

The concentration curve does more than offer a nice visual summary of wealth-based inequality in an indicator; it is also useful to quantify the degree of inequality revealed. The concentration index (*C*) quantifies the degree of inequality—twice the area between the concentration curve and the line of equality—which is analogous to how the Gini coefficient quantifies the degree of inequality in a Lorenz curve. We calculated C, for each indicator as follows:

3 $$ C=\frac{2}{\mu } COV\left(h,r\right) $$

where *h* is the health variable, μ is the mean, and *r* is the fractional rank of the individual in the wealth index.

When the outcome variable is binary, the concentration index has some questionable properties, especially comparing populations that have significantly different means. In particular, because it is mathematically bound between *μ − 1* and *1- μ* (where *μ* is the mean of the binary indicator), it tends to naturally fall (in absolute value) as the value of μ increases. If only the richest 10% have access to a health service in the base year, the concentration index would be 0.9. If the richest 90% have access to the health service in a subsequent year, the concentration index would be 0.1. It is therefore debatable whether that should be considered a large decrease in inequality [14, 15]. For binary indicators, we calculated as alternative indicators the Wagstaff concentration index [*W = 2/ μ (1- μ) COV(h, r)*] and the Erryegers concentration index, [*E = 8 COV(h, r)*], where *h* is a health outcome indicator and *r* is the fractional rank of the individual in the wealth index [14].

Our analysis also included decomposition of the concentration index measure of wealth-related inequalities in selected health outcomes and services. This allowed us to see how differences in, e.g., family size, women’s education, and access to safe water contribute to the observed wealth-related inequalities in health outcomes or health services, and how these patterns are changing over time. For example, the observed inequality in childhood vaccination rates might be explained entirely by differences in the mother’s education; decomposition of the concentration index would reveal that.

Decomposition of the concentration index is based on the algebraic transformation, which for a linear model of a health indicator

4 $$ y=\alpha +{\sum}_k{\beta}_k{x}_k+\varepsilon $$

allows the concentration index to be written as

5 $$ C={\sum}_k\left({\beta}_k{\overline{x}}_k/\mu \right)\ {C}_k+G{C}_{\varepsilon }/\mu $$

where μ is the mean of *y*, ${\overline{x}}_k$ is the mean of *x*_*k*_, *C*_*k*_ is the concentration index for *x*_*k*_, and *CG*_*ε*_ is the generalized concentration index for the error term [15].

The dependent variables of interest in this study are binary indicators and thus best modeled using a non-linear model such as probit. Following previous work [15, 16]), the linear approximation is given by the following specification,

6 $$ h={\alpha}^m+{\sum}_j{\beta}_j^m{x}_j+u $$

where *h* is the health variable of interest as defined earlier, *x*_*j*_ are the independent variables, *α* ^*m*^ is the constant term, ${\beta}_j^m$ are the partial effects of each variable treated as fixed parameters and evaluated as sample means, and *u* is the error term. The decomposed concentration index (C) for a health outcome *h*_*i*_ is therefore

7 $$ C={\sum}_j\left({\beta}_j^m\ {\overline{x}}_j/\mu \right){C}_j+{GC}_u/\mu $$

where *C*_*j*_ are the concentration indexes for *x*_*j*_, *μ* is the mean of the health variables *h*, ${\overline{x}}_j$ is the mean of *x*_*j*_, and *GC*_*u*_/*μ* is the residual component that captures inequality that is not explained by systematic variation in the regressors by income.

## Results

### Trends

Table 2 presents the profile and trends of selected MNCH outcomes and services. As the table makes clear, Ethiopia’s health service delivery is among the least developed in low-income countries—modern services reach only a small fraction of the population. For example, the results of the 2014 survey show that nationally modern contraceptive use is 45%. The situation is much worse for ANC visits, where services coverage was 25%, and SBAs, where coverage was 16%. Similarly, full immunization coverage in 2011 was 25%—among the lowest in similar countries in Sub-Saharan Africa. Over the last two decades, however, there has been considerable improvement in MNCH outcomes in Ethiopia, though child undernutrition and mortality rates are still high and coverage of maternal and health services is low. This holds true for all the health status and health service indicators analyzed in this study (Table 2): There is a consistent decline in ill health (undernutrition and mortality) and an increase in health services coverage (immunizations and maternal health services).

**Table 2:** Trends in MNCH outcomes in Ethiopia, 2000–2014

	2000	2005	2011	2014	Change (Latest-Earliest)
Stunting	57.0	49.5	44.1	40.6	−16.4
Wasting	12.5	12.4	10.1	8.9	−3.6
Underweight	41.9	34.1	29.1	26.6	−15.3
NNMR	48.4	39.3	37.4	34.3	−14.1
IMR	95.9	77.6	58.9	59.3	−36.6
U5MR	163.9	123.3	87.1	80.1	−83.8
Measles vaccination	27.0	36.5	56.7	-	29.7
Full immunization	14.6	21.6	24.9	-	10.3
Contraceptive	7.2	15.7	30.0	45.2	38.0
ANC4**+**	10.4	11.9	15.9	24.2	13.8
SBA	5.7	5.7	10.0	15.5	9.8

*Source*: Authors’ compilation from the EDHS (2000, 2005, 2011 & 2014) data

Notes: The values are number of births per 1000 live births for NMR, IMR and U5MR and percentages for the rest. Earliest survey year is 2000 for all indicators. Latest survey year is 2014 for all but full immunization and measles vaccination, for which our latest source of information is the 2011 DHS

**Fig. 1:**
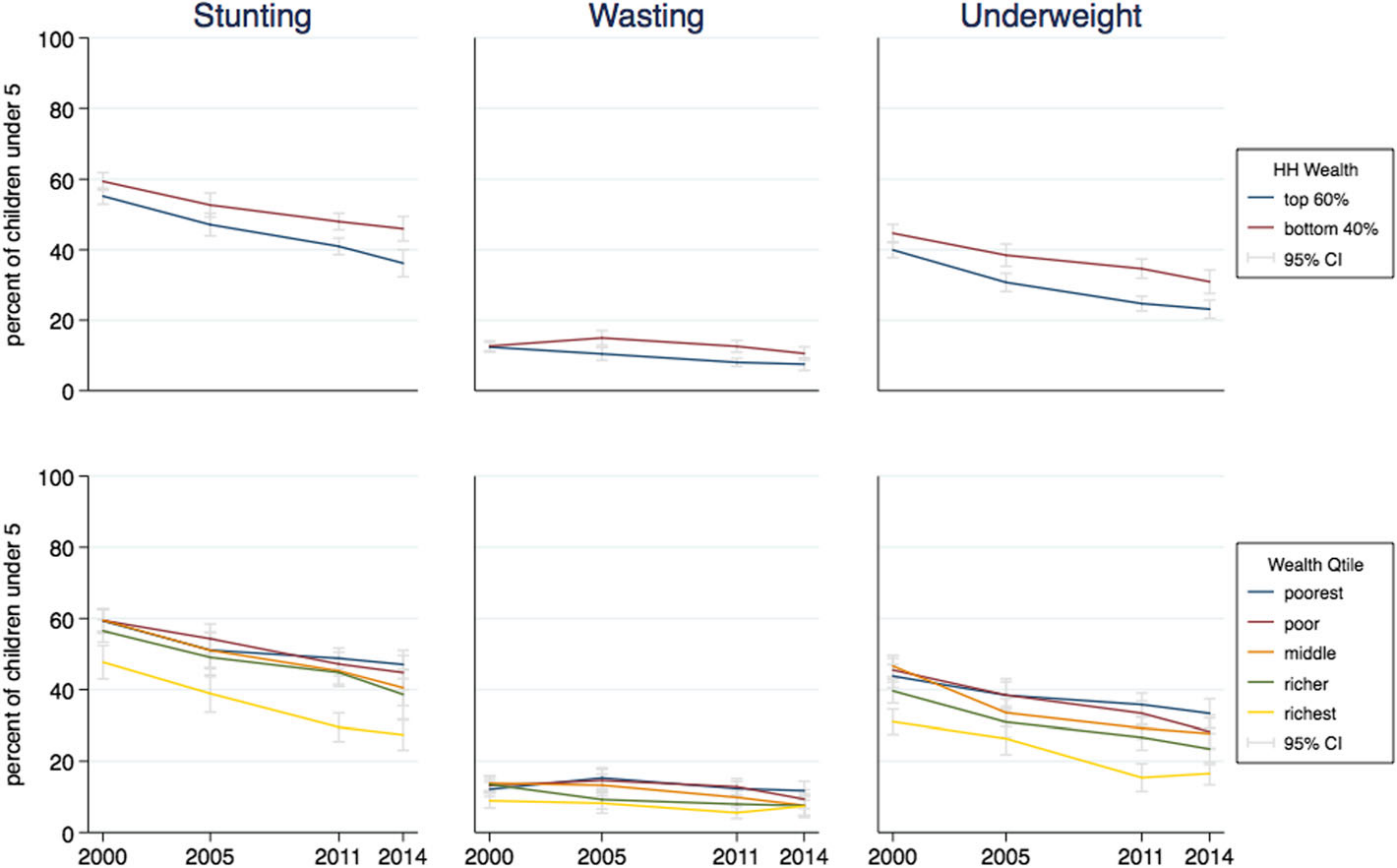
Trends in child malnutrition by wealth ranking, 2000–2014. *Source:* DHS 2000 and mini-DHS 2014 data. *Notes:* Percent of children under 5 years old. Grey lines represent 95% confidence intervals

**Fig. 2:**
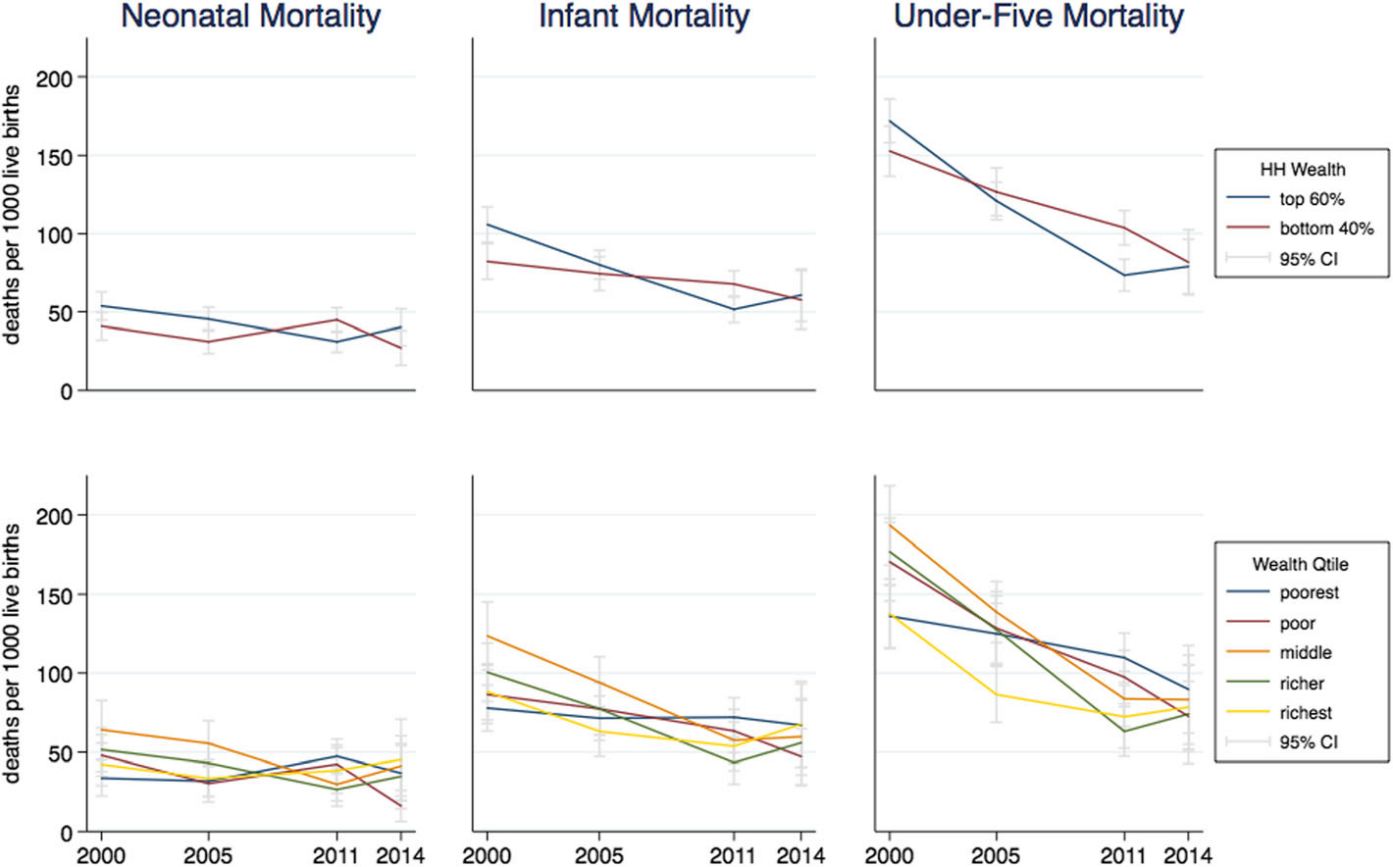
Trends in child mortality by wealth ranking, 2000–2014. *Source:* DHS 2000 and mini-DHS 2014 data. *Notes:* Deaths per 1000 live births. Grey lines represent 95% confidence intervals

**Fig. 3:**
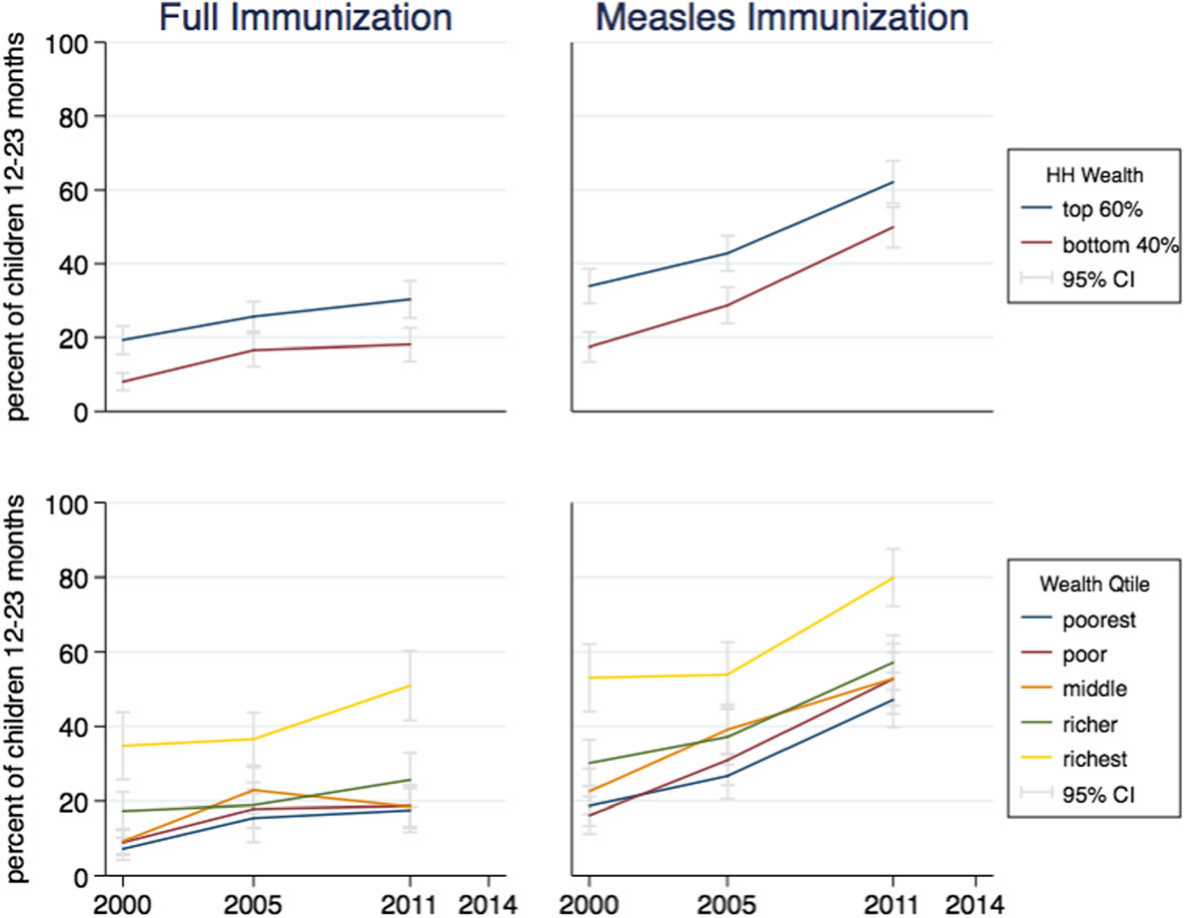
Trends in child immunization by wealth ranking, 2000–2011. *Source:* DHS 2000 and 2011 data. *Notes:* Percent of children 12–23 months old. Grey lines represent 95% confidence intervals

**Fig. 4:**
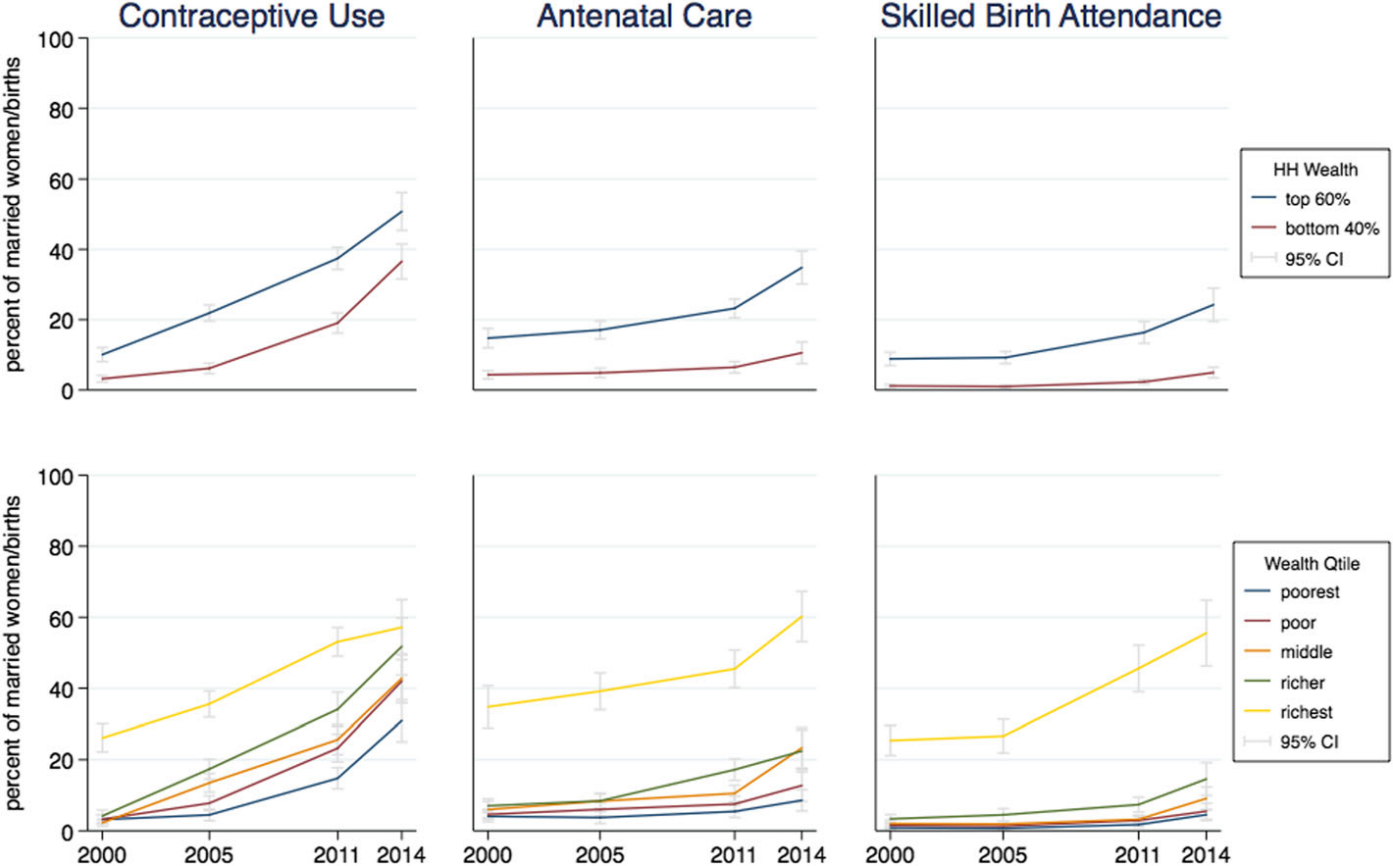
Trends in maternal health services by wealth ranking, 2000–2014. *Source:* DHS 2000 and mini-DHS 2014 data. *Note*: Percent of married women/births. Grey lines represent 95% confidence intervals

To look at the trends by wealth status, we disaggregated the progress made. For each indicator, each line represents the value of the indicator for one group over time, with the 95% confidence interval around each value. Figures 1 to 4 present the results for child nutrition, child mortality, immunization, and maternal health services. As expected, as a general pattern the lines for adverse outcomes slope downward for all groups and the lines for health services slope upward, showing MNCH is improving at all wealth levels; the improvements in average national figures, however, do not hide worsening results for the poor. The distances between the curves and the slope of each curve in each graph show differing initial and final inequality for the indicators.

In Fig. 1, the lines in all graphs move downward basically in parallel, with perhaps a slight widening of the gap between the different wealth groups. In the graph for stunting, we see that inequality is driven by the difference between the richest quintile and the rest, and that this difference is increasing slightly.

Figure 2 shows the trends for child mortality indicators. The biggest improvement has been in the U5MR, with modest improvements in the IMR and essentially no change in neonatal mortality. No clear pattern of wealth-related inequality is observable. The confidence intervals often overlap, showing no significant difference between different groups, and the lines crisscross each other, with poorer households sometimes seeming to have lower child mortality (which might be true and reflect differences in practices like breastfeeding). It is not clear whether it is actually true that there is no systematic difference in child mortality by household wealth level or whether the figures are obscured by the data collection strategy (which only counts children whose mother is alive) or cultural traditions (unwillingness to speak about an infant who died very young).

In Fig. 3, the trends show that immunization services have expanded for all wealth groups, with the lines moving roughly in parallel. Again, the biggest gap is between the richest quintile and all the others.

As Fig. 4 shows, maternal health services have also improved for all groups, but there is a widening of the gap between rich and poor, with a greater increase in the use of maternal health services by richer households. This trend is less pronounced for contraception use, and in fact there seems to be a slight closing of the gap in the last year of data. Again, we see that wealth-based inequality is primarily driven by the difference between the wealthiest quintile and the rest, especially for ANC and SBA.

### Absolute and relative inequalities

The figures in the previous section give us a good indication of the general trends: fairly consistent improvements across all wealth groups, with some modest narrowing or widening of gaps depending on the indicator and the exact breakdown of households by wealth. To quantify and test the statistical significance of these observations, we report the results from eqs. 1 and 2 in Tables 3 and 4. The results in Table 3 compare the bottom 40% of households and the top 60%. Those in Table 4 compare the bottom quintile to the top quintile. In each table, the first section gives the value of the indicator for “poor” and “rich” households in both the first and the last year. The second shows the absolute inequality (the difference between the values of the indicator for the two groups) in the earliest and the latest survey and the *p*-value for whether absolute inequality changed significantly during that period. The third section shows the relative inequality (the ratio of the indicators) and the *p*-value for whether it has changed significantly. A positive difference or ratio greater than 1 for an ill-health outcome (child undernutrition or child mortality) shows a pro-rich inequality—child undernutrition and mortality rates were lower for children from better-off households. Likewise, a negative difference or ratio less than 1 in any of the immunization and maternal health service indicators (good health service utilization) implies pro-rich inequality. These socioeconomic differences are to be expected; our interest is in whether the differences are increasing or decreasing.

**Table 3:** Trends in MNCH between poor and rich households, 2000–2014

	Top 60%		Bottom 40%		Difference			Ratio		
	Earliest Survey	Latest Survey	Earliest Survey	Latest Survey	Earliest Survey	Latest Survey	*p*-value	Earliest Survey	Latest Survey	*p*-value
Stunting	55.2	36.2	59.4	45.9	−4.2	−9.8	0.064	0.9	0.8	0.013
Wasting	12.4	7.5	12.7	10.6	−0.3	−3.0	0.077	1.0	0.7	0.034
Underweight	39.9	23.1	44.7	30.9	−4.7	−7.8	0.189	0.9	0.7	0.011
NNMR	53.8	40.3	40.9	27.1	12.9	13.2	0.977	1.3	1.5	NA
IMR	105.8	60.7	82.2	57.7	23.6	3.0	0.176	1.3	1.1	NA
U5MR	171.9	79.0	152.7	81.7	19.2	−2.7	0.212	1.1	1.0	NA
Measles vaccination	33.9	62.1	17.5	49.9	16.5	12.2	0.354	1.9	1.2	0.006
Full immunization	19.3	30.3	8.1	18.1	11.2	12.2	0.796	2.4	1.7	0.143
Contraceptive	10.1	50.8	3.2	36.6	6.9	14.2	0.033	3.2	1.4	0.002
ANC4+	14.8	34.8	4.3	10.6	10.4	24.2	0.000	3.4	3.3	0.876
SBA	8.9	24.2	1.2	5.0	7.7	19.3	0.000	7.5	4.9	0.142

*Source*: Authors’ compilation from the EDHS (2000, 2005, 2011 & 2014) data

*Notes*: Indicators are given in percentage points except for mortality rates, which are number of deaths per 1000 live births. The earliest year is 2000 for all indicators. The latest year is 2014 for all indicators except immunization (full and measles), for which it is 2011

**Table 4:** Trends in MNCH between poorest and richest households, 2000–2014

	Richest Quintile		Poorest Quintile		Difference			Ratio		
	Earliest Survey	Latest Survey	Earliest Survey	Latest Survey	Earliest Survey	Latest Survey	*p*-val	Earliest Survey	Latest Survey	*p*-val
Stunting	47.7	27.4	59.3	47.1	−11.6	−19.7	0.057	0.8	0.6	0.002
Wasting	8.9	7.4	12.1	11.7	−3.2	−4.3	0.656	0.7	0.6	0.559
Underweight	31.0	16.6	43.8	33.4	−12.8	−16.8	0.237	0.7	0.5	0.003
NNMR	42.3	45.3	33.7	36.8	8.6	8.4	0.991	1.3	1.2	NA
IMR	88.2	67.7	77.9	67.0	10.3	0.7	0.669	1.1	1.0	NA
U5MR	137.6	78.4	136.1	89.7	1.5	−11.3	0.603	1.0	0.9	NA
Measles vaccination	53.1	79.9	18.7	47.1	34.3	32.8	0.825	2.8	1.7	0.023
Full immunization	34.8	50.9	7.2	17.5	27.6	33.4	0.419	4.8	2.9	0.153
Contraceptive	26.1	57.2	3.2	30.9	23.0	26.2	0.538	8.3	1.8	0.000
ANC4**+**	34.8	60.2	4.1	8.6	30.7	51.7	0.000	8.6	7.0	0.481
SBA	25.4	55.6	0.9	4.5	24.5	51.0	0.000	29.6	12.3	0.060

*Source*: Authors’ compilation from the EDHS (2000, 2005, 2011 & 2014) data

*Notes*: Indicators are given in percentage points except for mortality rates, which are number of deaths per 1000 live births. The earliest year is 2000 for all indicators. The latest year is 2014 for all indicators except immunization (full and measles), for which it is 2011

The results (Table 3) point to a widening of absolute pro-rich inequality in child nutritional outcomes between the poor (bottom 40%) and the rich (top 60%)—an inequality observed in all three child nutrition status indicators but more significant for stunting and underweight. For the poor, child stunting in the earliest survey was higher by about 4.2 percentage points and in the latest the difference rises to 10 percentage points. Similarly, the gap in underweight went up from 5 to 8 percentage points. In both cases, these changes are statistically significant, which implies that pro-rich inequality is widening. The same conclusions can be drawn when looking at relative inequalities: pro-rich inequality widened significantly during the period considered, at least for stunting and underweight.

When we restrict our analysis to the poorest versus the richest quintile (Table 4), the trend recurs: an ever-increasing gap between the rich and the poor in terms of child nutrition. These changes are less statistically significant, however, and only the relative inequality in stunting between the richest and the poorest changes significantly.

The results for infant and child mortality appear to show that mortality rates were in fact lower for poorer households in the first survey (the values for the difference between rich and poor are negative in all three indicators and in both ways of defining rich and poor). Then, by the final survey, the pro-poor gap appears to have disappeared for infant mortality and is reversed for under-5 mortality, with both rates higher for poorer households. While these figures may represent real changes in mortality patterns, it should be noted that the standard errors on the mortality rates for different subgroups are quite high, and that none of the changes over time are statistically significant.

For all services except measles vaccination, in the first survey, utilization by poorer households was very low; thus while the *difference* between the rates increased (significantly in the case of maternal health services), which suggests widening inequality, the *ratio* of the rates decreased, suggesting that inequality had narrowed. For measles vaccination, both the variances and the ratio suggest decreasing inequality, although only the changes in the ratio are significant.

### Concentration curves and indexes

**Fig. 5:**
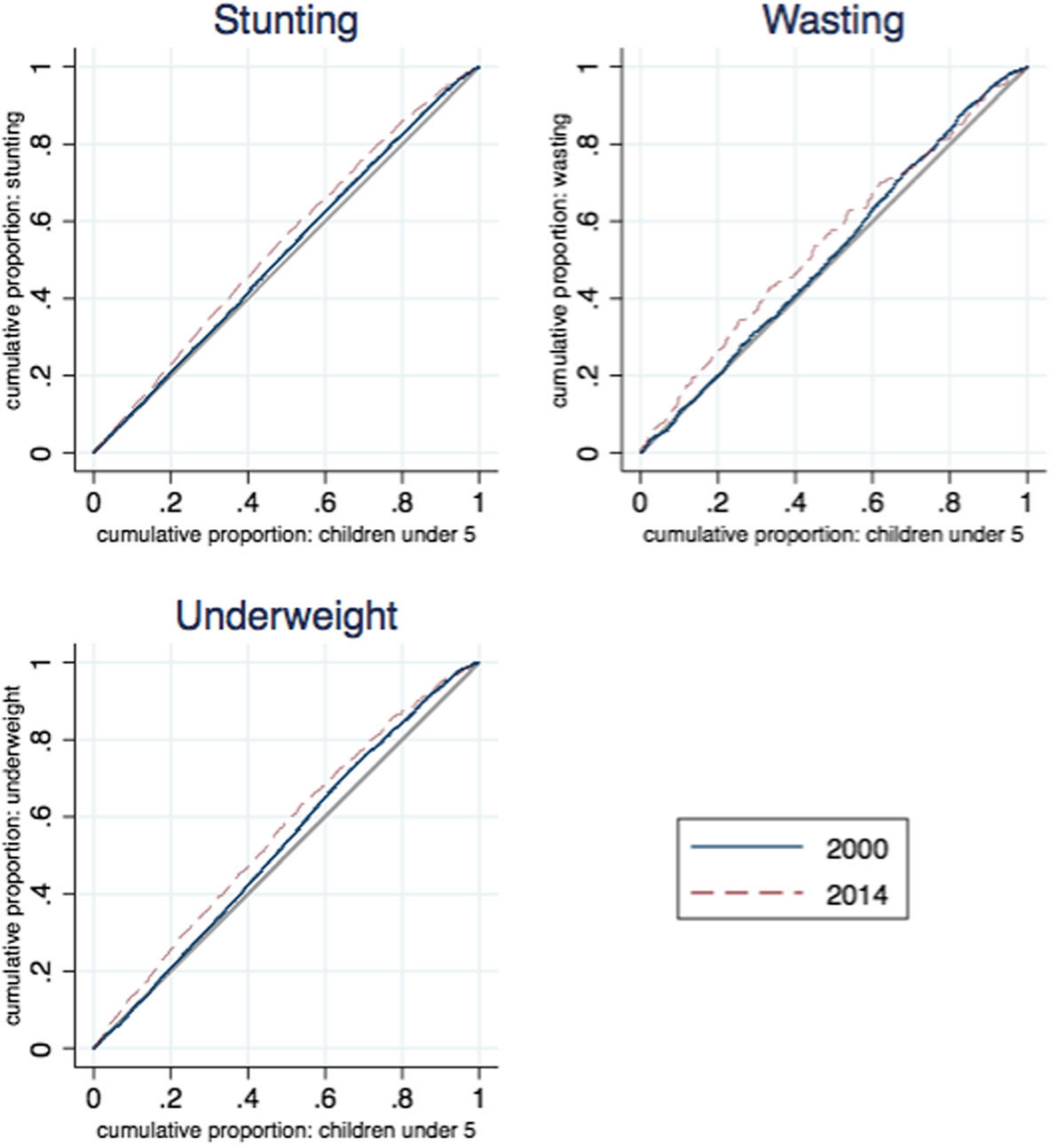
Concentration curves of child nutritional status, 2000–2014. *Source:* DHS 2000 and mini-DHS 2014 data. *Note:* Percent of children under 5 years old

The concentration curves in Fig. 5 show child malnutrition as fairly even distributed across income categories, with the curves lying close to the line of equality, but the curves moving away from the line indicate gradually increasing inequality. Overall, the results presented here are aligned with the findings already presented: the poor did not benefit as much from the improvements in child nutritional status.

**Fig. 6:**
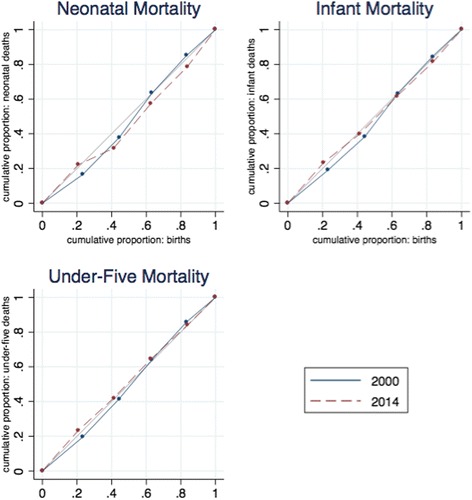
Concentration curves of child mortality, 2000–2011. *Source:* DHS 2000 and mini-DHS 2014 data

Figure 6 shows the concentration curves for child mortality indicators. Here the curves overlap and some cross the line of equity. This reflects the fact that there are no obvious patterns or trends when mortality rates are broken down by household wealth level. Statistical tests confirm that for these indicators there is no significant change in wealth-based inequality.

**Fig. 7:**
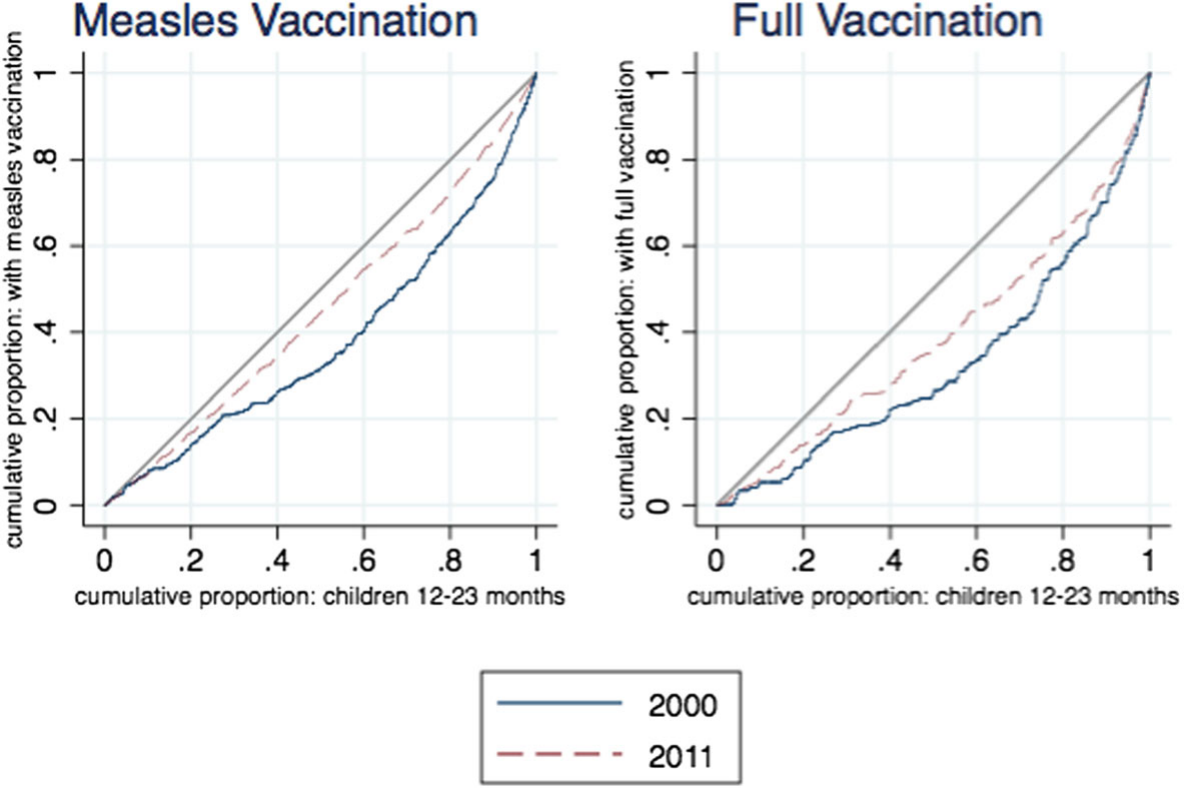
Concentration curves of child immunization coverage, 2000–2011. *Source:* DHS 2000 and 2011 data. *Notes:* Percent of children 12–23 months old

**Fig. 8:**
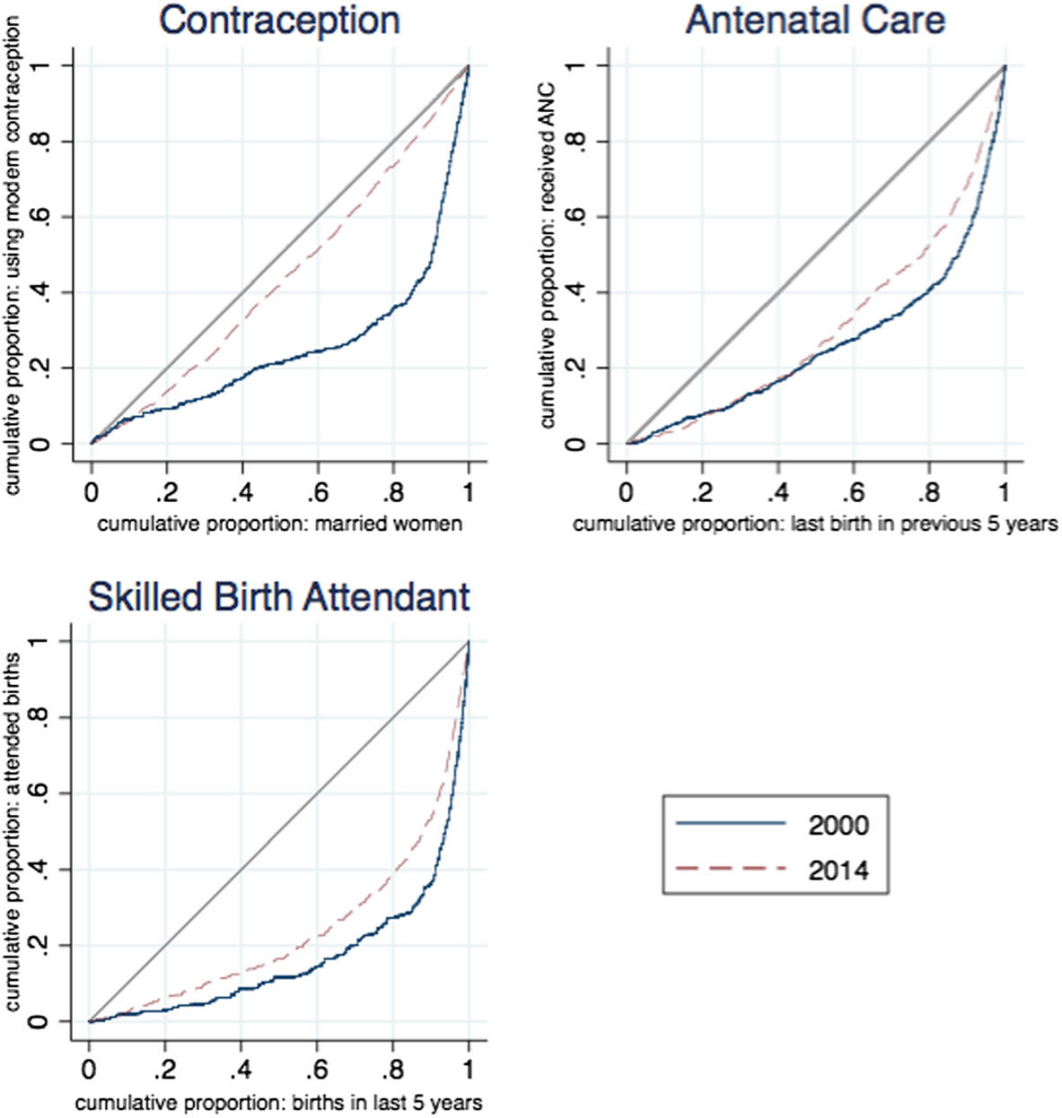
Concentration curves of selected maternal health services, 2000–2014. *Source:* DHS 2000 and mini-DHS 2014 data

Figs. 7 and 8 illustrate the movement of wealth-related inequalities in child immunizations and maternal health services. Here there is clearly significant inequality, with the concentration curves lying far below the line of equality. However, we also see substantial movement over time as the curves move closer to the line of equality, reflecting less wealth-based inequality in use of services. The figure for antenatal care shows clearly that improvements are concentrated among richer households— middle-ranked households are catching up to the wealthiest households, but the poorest households are not catching up to those in the middle.

For each indicator, Table 5 provides information on the concentration index and, for binary indicators, the alternative Wagstaff and Erryegers concentration indexes for the earliest and latest years. Note that, as expected, the index values are negative for malnutrition indicators—richer households have less malnutrition—and positive for MNCH services—richer households use health services more. The values of the concentration indexes for mortality can be either positive or negative; there is no clear trend. The absolute value of the concentration index can be taken as a measure of the inequality present. The tables also show the result of simple t-tests of whether the concentration index value changed significantly between the first and the last survey.

**Table 5:** Concentration indexes of selected child health status indicators in Ethiopia, 2000–2014

	Concentration Index			Wagstaff Concentration Index			Erryegers Concentration Index		
	Earliest	Latest	*p*-val	Earliest	Latest	*p*-val	Earliest	Latest	*p*-val
Stunting	−0.032	−0.086	0.001	−0.075	−0.145	0.018	−0.074	−0.140	0.021
Wasting	−0.034	−0.102	0.105	−0.039	−0.112	0.116	−0.017	−0.036	0.240
Underweight	−0.054	−0.119	0.003	−0.093	−0.162	0.025	−0.091	−0.127	0.163
NNMR	0.049	0.075	0.000	0.052	0.078	0.000	−	−	−
IMR	0.038	0.008	0.000	0.042	0.009	0.000	−	−	−
U5MR	0.016	−0.022	0.000	0.019	−0.024	0.000	−	−	−
Measles vaccination	0.242	0.096	0.000	0.331	0.223	0.043	0.261	0.219	0.368
Full immunization	0.344	0.223	0.025	0.403	0.297	0.110	0.201	0.22	0.594
Contraceptive	0.496	0.119	0.000	0.534	0.218	0.000	0.142	0.216	0.009
ANC4+	0.474	0.381	0.017	0.529	0.503	0.578	0.197	0.369	0.000
SBA	0.665	0.525	0.004	0.705	0.622	0.116	0.150	0.325	0.000

*Source*: Authors’ compilation from the EDHS (2000, 2005, 2011 & 2014) data

*Note*: Latest survey for immunization indicators is 2011

The concentration indexes for all three undernutrition indicators show pro-rich inequality heightening; in absolute value the concentration index more than doubled for all three, although from initially low levels of inequality. The changes are significant for stunting and underweight. Using the Wagstaff or the Erryegers concentration index yields the same result but with somewhat less significance. On the other hand, like the concentration curves (Fig. 5), there is no clear pattern in the indexes for mortality indicators (Table 5). Table 5 also shows mixed progress in decreasing inequality in the use of MNCH services; over time concentration index values decrease, but except for measles vaccination and contraceptive use the changes are not significant when the Wagstaff concentration index is used. However, the Erryegers concentration index leads to the opposite conclusions: it shows increasing inequality in four of the five indicators and is significant for maternal health services. This again reflects the pattern we see, particularly in maternal health services: having expanded rapidly from low levels tends to lead to a decrease in the standard concentration index that is not robust to using the Erryegers index.

Despite progress then, as both the concentration curves and indexes illustrate, inequalities are still substantial in the case of ANC4+ and SBA.

### Changes in inequality

We used three different methods to assess changes over time in wealth-related inequality in indicators of health status and health service utilization: rate differences, rate ratios, and concentration indices. Table 6 summarizes the results.

**Table 6:** Summary of results of changes in income related MNCH inequalities, 2000–2014

	Rate Differences		Rate Ratio		Conc. Index	Test of Dominance: Concentration Curves	
	Bottom 40% vs. Top 60%	Poorest (q1) vs. Richest (q5)	Bottom 40% vs. Top 60%	Poorest (q1) vs. Richest (q5)		mca rule	iup rule
*Panel A: Health Status*							
Stunting	Worsened	Worsened	Worsened	Worsened	Worsened	NS	NS
Wasting	Worsened	NS	Worsened	NS	NS	Worsened	NS
Underweight	NS	NS	Worsened	Worsened	Worsened	Worsened	NS
NMR^a^	NS	NS	−	−	Improved^a^	−	−
IMR^a^	NS	NS	−	−	Improved^a^	−	−
U5MR^a^	NS	NS	−	−	Improved^a^	−	−
*Panel B: Health Services*							
Measles vaccination	NS	NS	Improved	Improved	Improved	Improved	NS
Full immunization	NS	NS	NS	NS	Improved^b^	NS	NS
Contraceptive	Worsened	NS	Improved	Improved	Improved	Improved	NS
ANC4+	Worsened	Worsened	NS	NS	Improved^b^	Improved	NS
SBA	Worsened	Worsened	NS	Improved	Improved^b^	Improved	Improved

*Notes*: The table summarizes results of different approaches presented in the previous sections and the test of dominance. The test of dominance is based on [15]. The number of evenly spaced quintile points is 19 (from 5% to 95%) and the significance level is 5%. The dominance test rules, mca and iup, respectively denote the multiple comparison approach and the intersection union principle. “–” is test not applicable. NS is no significant change from the earliest survey

^a^The improvement for mortality indicators is a progression of the distribution from a more pro-poor towards the line of equality. ^b^Improvement is not significant if Wagstaff concentration index is used

Although child malnutrition has been reduced for all income groups, wealth-based inequality worsened over the period studied. This pattern is consistent for all three indicators and all methods of analysis.

The DHS data show no clear relationship between household wealth and child mortality. In earlier years, there may have been inequality in favor of poorer households, or possibly an inverse-U shaped pattern, with households in the middle of the wealth distribution having the highest child mortality. That pattern might be explained by cultural factors, or it might be an artifact of the data collection strategy. The concentration index suggests there is some evidence that inequality in child mortality has “improved,” moving from a pro-poor bias to a smaller pro-poor bias or a small pro-rich bias.

Inequality has narrowed slightly in child vaccination services, particularly for measles vaccinations. For maternal health services, the results seem contradictory: The absolute difference in maternal health service utilization by the rich and the poor has increased, but initially service use by poor households was extremely low and the gains they have made are substantial. Thus, the rate ratio, the concentration curves, and two of the three concentration indexes analyzed all rate the changes as a decrease in inequality.

### Decomposition

The analyses so far discussed demonstrate that, despite improvements, there are still considerable inequalities in some MNCH outcomes and services. In this section, we examine what may be contributing to the wealth-related inequalities in certain indicators.

Tables 7 to 9 present the decomposition of concentration indexes for selected indicators. Various controls that may be related to both wealth status and the value of the indicator are included to see how much of the inequality can be attributed to factors like the mother’s education or adequate sanitation facilities.

**Table 7:** Decomposition of concentration index for stunting (2000–2014)

	2000			2014		
	Elasticity	Conc. index	Contribution	Elasticity	Conc. index	Contribution
Child age	1.639	−0.003	−0.004	2.202	−0.011	−0.025
Child age squared	−0.862	−0.004	0.003	−1.168	−0.016	0.018
Sex (Male)	0.028	−0.004	0.000	0.031	0.010	0.000
Birth order	0.010	−0.068	−0.001	−0.048	−0.024	0.001
Wealth index	−0.192	0.142	−0.027	−0.310	0.180	−0.056
Child HH members	0.038	−0.016	−0.001	0.000	−0.033	0.000
HH size	−0.008	−0.025	0.000	0.057	0.005	0.000
Education (mother)	−0.020	0.531	−0.011	−0.037	0.460	−0.017
Education (HH head)	−0.018	0.347	−0.006	−0.026	0.349	−0.009
Residence (urban)	0.010	0.848	0.009	−0.013	0.709	−0.009
Region	.	.	−0.005	.	.	−0.001
Residual	.	.	0.010	.	.	0.055
Total	.	.	−0.033	.	.	−0.042

*Notes*: Child HH members are household members under 5 years of age. HH size is total household size. Regions are dummies for each region with one reference region (Tigray) omitted from the regressions

Table 7 summarizes the results for stunting: in each year, even when controlling for other variables, the largest contribution comes from the wealth index. The next largest comes from mother’s education. Comparing the progression of stunting between 2000 and 2014, we see that the increased inequality comes only from the contribution of the wealth index itself, not from any other factors. The decomposition for 2014 has a large positive residual component, however, suggesting that other factors not captured in the decomposition are in fact offsetting the rise in wealth-related inequality in stunting.

Table 8 shows the decomposition for measles vaccination (Panel a) and full immunization (Panel b). Comparing results in 2000 and 2014, we see that for measles vaccination the contribution of the wealth index has gone from being the largest to being one of the smallest but when all immunizations are considered it was still very important. This suggests that the pure wealth component of inequality in measles vaccinations almost disappeared when coverage was expanded, as it was for measles vaccination but not full immunization. In both cases, the remaining inequalities can be explained almost entirely by differences in the education of the mother and the father.

**Table 8:** Decomposition of concentration index for immunization indicators (2000–2011)

	2000			2011		
*8a. Measles Vaccination*	Elasticity	Conc. ind	Contribution	Elasticity	Conc. ind	Contribution
Age (mother’s)	0.124	−0.023	−0.003	0.304	0.000	0.000
Child HH members	−0.011	−0.014	0.000	−0.073	−0.030	0.002
HH size	−0.036	−0.027	0.001	−0.109	−0.005	0.001
Wealth index	1.437	0.098	0.141	0.856	0.104	0.089
Education (mother)	0.057	0.518	0.029	0.070	0.486	0.034
Education (father)	0.107	0.407	0.043	0.024	0.364	0.009
Residence (urban)	−0.006	0.885	−0.005	−0.011	0.800	−0.009
Region	.	.	0.004	.	.	−0.001
Residual	.	.	0.031	.	.	−0.029
Total	.	.	0.242	.	.	0.096
*8b. Full Immunization*						
Age (mother’s)	0.096	−0.023	−0.002	0.362	0.000	0.000
Child HH members	0.015	−0.014	0.000	−0.091	−0.030	0.003
HH size	0.065	−0.027	−0.002	−0.207	−0.005	0.001
Wealth Index	1.409	0.098	0.138	2.434	0.104	0.253
Education (mother)	0.110	0.525	0.058	0.042	0.486	0.021
Education (father)	0.112	0.412	0.046	0.000	0.363	0.000
Residence (urban)	−0.021	0.906	−0.019	−0.065	0.800	−0.052
Region	.	.	.	.	.	−0.005
Residual	.	.	.	.	.	0.002
Total	.	.	0.344	.	.	0.223

*Notes*: Child HH members are household members under 5 years of age. HH size is total household size. Regions are dummies for each region with one reference region (Tigray) omitted from the regressions

In Table 9 Panels a through c present the decomposition results for maternal health services. A serious limitation is that in all specifications the residual component is large. With that in mind, however, it is still possible to discern some common trends: there is only a slight decline in inequalities over the period, the prevailing inequalities are still high, and the wealth index, education, and residence of the user all make important contributions to the differences in utilization of these services.

**Table 9:** Decomposition of concentration index for maternal health services (2000–2014)

	2000			2011/14		
*9a. Contraceptive*	Elasticity	Conc. index	Contribution	Elasticity	Conc. index	Contribution
Age	−0.481	−0.019	0.009	−0.768	−0.006	0.004
Wealth index	1.028	0.099	0.102	0.780	0.101	0.079
Education (years)	0.067	0.562	0.038	0.062	0.432	0.027
Residence (urban)	0.152	0.817	0.124	0.067	0.636	0.043
Protestant	−0.014	−0.044	0.001	0.013	−0.019	0.000
Muslim	0.046	−0.018	−0.001	−0.073	−0.041	0.003
No more children	0.275	0.020	0.006	0.096	0.002	0.000
Region	.	.	−0.009	.	.	0.003
Residual	.	.	0.227	.	.	0.224
Total	.	.	0.496	.	.	0.382
*9b. ANC4+*						
Age	0.102	−0.020	−0.002	−0.404	−0.005	0.002
Wealth index	1.430	0.100	0.143	1.764	0.130	0.230
Education (years)	0.103	0.566	0.058	0.167	0.449	0.075
Residence (urban)	0.078	0.851	0.066	0.064	0.708	0.046
Region	.	.	0.021	.	.	0.022
Residual	.	.	0.186	.	.	0.092
Total	.	.	0.474	.	.	0.467
*9c. SBA*						
Age	−0.085	−0.019	0.002	−0.46	−0.002	0.001
Child HH members	−0.33	−0.023	0.008	−0.476	−0.037	0.018
HH size	0.124	−0.029	−0.004	−0.324	0.005	−0.002
Wealth index	1.278	0.095	0.122	2.3	0.125	0.287
Education (years)	0.096	0.53	0.051	0.161	0.441	0.071
Residence (Urban)	0.087	0.856	0.074	0.06	0.681	0.041
Region	.	.	0.023	.	.	0.001
Residual	.	.	0.389	.	.	0.108
Total	.	.	0.665	.	.	0.526

*Notes*: Child HH members are household members under 5 years of age. HH size is total household size. The latest survey for ANC4+ and SBA is 2014. The latest survey used in this table for Contraceptive is 2011. We used 2011 to add more family planning variables that were not included in the 2014 mini-DHS. Regions are dummies for each region with one reference region (Tigray) omitted from the regressions

## Discussion

In examining differential progress in health status and health services utilization in Ethiopia, we used a variety of methods to look at inequalities by household wealth in selected MNCH indicators. We are able to make three main observations:

1. According to trend analysis, there has been substantial progress in MNCH services and outcomes over the study period. DHS data for 2000–14 show that child undernutrition and mortality have declined considerably and health services coverage has increased. Indeed, Ethiopia is one of the few countries that achieved its MDG4 (child mortality reduction) three years ahead of schedule, by three years. As in many other countries, that may partly be due to its economic performance during the past decade and the related improvements in living conditions. A recent poverty assessment study found that in 11 years head-count poverty dropped by 24 percentage points, from 55% in 2000 to 31% in 2011 [17]. Ethiopia’s health system may also have made an indispensable contribution. In the period from 1997 to 2015, the Health Sector Development Programs (HSDP) allocated resources to priority health outcomes and made services locally available through power devolution and expansion of infrastructure and human resources [18].
2. The results of the analyses of wealth-related inequalities are mixed. Over time, there has been a narrowing of wealth-based inequalities in health services (measles and full vaccination, contraception, ANC4+, and SBA). This is encouraging. It suggests that further expansion in their coverage could substantially reduce the remaining inequalities. However, we also find that for the poor, health outcomes have worsened, and the gap in child malnutrition between rich and poor households has widened. Patterns in childhood mortality may also have shifted from a modest pro-poor bias to a modest pro-rich one. This finding on the disconnect between health services and health outcomes agrees with previous studies for a number of developing countries [4]. However, the considerable decline in health services inequality could be attributable to the country’s flagship Health Extension Program, which may have helped significantly to making services more available, particularly to the poor. The program deployed over 38,000 health extension workers (HEWs) to local communities for health promotion and basic service delivery [19]. As a lower-cadre alternative in their own or neighboring communities, these HEWs reached poor Ethiopians more effectively than medical doctors and nurses. However, because improving health and nutritional outcomes among the poor requires coordinated action across the health, education, agriculture, and water sectors [20–22], it may take time to reduce the related inequality.
3. Despite the narrowing trend, there is still substantial inequality in health services, especially in ANC4 and SBA. For example, in the 2014 survey, 35% of the top 60% made four or more ANC visits compared to 11% of the poorer 40%. Similarly, 24% of women from the top 60% of households had SBA, compared to just 5% of women from the bottom 40%. In general, differences in favor of the rich (top 60%) are large for most of the outcomes studied. The differences are even more pronounced between the poorest quintile (bottom 20%) and the richest (top 20%).

The correlations observed between household wealth and child health outcomes or MNCH service use are driven in part by such intermediate factors as women’s education level and access to services like water and sanitation. However, the decomposition of inequalities found that even once these factors are accounted for, household wealth still has a direct effect on health service use and outcomes, and the changes in inequality observed are driven mainly by the direct effects rather than the intermediate factors [21, 22]. Increasing coverage of ANC4 and SBA goes beyond just making services available; there are also concerns about the quality of services (e.g., qualified staff, equipment, water and electricity for laboratories and delivery). The Government of Ethiopia has started taking action in this area, for example upgrading HEW qualifications so that they can be the equivalent of midwives. There have also been efforts to inventory facility readiness and address bottlenecks. Over the long term, a continuous quality improvement system could help to narrow the inequalities in health outcomes.

## Limitations

How the DHS is designed affects how the indicators are constructed in ways that may affect the analysis of inequality. For example, because data on child vaccinations are collected as part of the women’s questionnaire, they are only available for children whose biological mother is alive and in the household. Similarly, because infant and child mortality rates are calculated from information collected from women about all their births in the previous five years, it does not take into account deceased children whose mothers died giving birth or subsequently. In contrast, because data on height and weight were collected for all the children in the household, they should be fully representative.

In the analysis, we do see significantly worse nutrition outcomes for children whose biological mother is not alive or not in the household, suggesting that mortality rates may be underestimated and immunization rates overestimated. These biases would be worse for subgroups with higher rates of maternal mortality or (in the case of immunizations) fostering; if the biases are large, they may skew the inequality analysis.

Another limitation is that because data on ANC and SBA are collected in the women’s questionnaire, they do not include pregnancies and births that ended in the death of mother. We would expect that poor antenatal or delivery care would be a risk factor for maternal mortality; we could be overestimating ANC and SBA, again with possibly differential biases based on the rates of maternal mortality in various subgroups.

## Conclusions

Ethiopia’s recent progress in MNCH was the starting point for fuller examination of the trends for rich and poor, and how wealth-related health inequality changed over the last two decades. The results obtained using various approaches led to similar conclusions: We found pro-rich inequality in certain health status outcomes but in general pro-poor progress in services. In addition, in both health status and services, there is still substantial wealth-related inequality. The decomposition exercise shows how certain socioeconomic status indicators, such as the wealth index and education, may help to explain existing inequalities. Ethiopia’s efforts to improve access to health services have shown some positive results, but now it may be necessary, to change outcomes for the poorest, to focus on service quality and cross-sectoral interventions.

## Additional file

Additional file 1: Table S1a. Detailed Definition of Indicators. **Table S1b.**Definition of Terms. (DOCX 58 kb)

## Abbreviations

ANC4+: Antenatal care, four or more visits
CPR: Contraceptive prevalence rate
DHS: Demographic and Health Survey
HEW: Health extension worker
IMR: Infant mortality rate
IUD: interauterine device
iup: intersection union principle (dominance test)
mca: multiple comparison approach (dominance test)
MDGs: Millennium Development Goals
MNCH: Maternal, newborn, and child health
NMR: Neonatal mortality rate
SBA: Skilled birth attendant
SDGs: Sustainable Development Goals
U5MR: Under-5 mortality rate
VCHW: Voluntary community health worker
