# Supplementary material for: Examining changes in maternal and child health inequalities in Ethiopia
Source: Int J Equity Health. 2017 Aug 22;16:152. doi: 10.1186/s12939-017-0648-1 (PMC5568328; doi:10.1186/s12939-017-0648-1)
Supplement: Additional file 1: Table S1a. — Detailed Definition of Indicators. Table S1b.Definition of Terms. (DOCX 58 kb) [file 12939_2017_648_MOESM1_ESM.docx]

Additional file 1

Table S1a Detailed Definition of Indicators

| Indicator | Defined for | Equal to 1 if |
| --- | --- | --- |
| Stunted | All children 0-59 months old living in the household * | Child has height-for-age z score < -2 ** |
| Wasted | All children 0-59 months old living in the household * | Child has weight-for-height z score < -2 ** |
| Underweight | All children 0-59 months old living in the household * | Child has weight-for-age z score < -2 ** |
| Full immunization | All children 12-23 months old born to women currently aged 15-49 and living in household | Child received BCG, measles and three doses each of polio and DPT at any time, verified either by card or recall of mother |
| Measles vaccination | All children 12-23 months old born to women currently aged 15-49 and living in household | Child received measles vaccine at any time, verified either by card or recall of mother |
| SBA | All births in last 5 years to women currently aged 15-49 living in the household | Birth was attended by skilled health attendant + |
| ANC4+ | Most recent birth for women aged 15-49 living in the household who gave birth in past 5 years | Woman received four or more antenatal care visits by skilled health personnel + |
| Contraceptive | Married women ages 15-49 living in the household who aren’t sure they are pregnant | Woman currently uses a modern form of contraception ++ |
| NNMR | Neonatal mortality rate using data for the 5 years preceding the survey # | |
| IMR | Infant mortality rate using data for the 5 years preceding the survey # | |
| U5MR | Under-5 mortality rate using data for the 5 years preceding the survey # | |

Notes: * In 2005, only half the households were selected for child weighing/measuring.

** 20006 WHO Child Growth Standards

+ See table S1b for year by year definitions of “skilled health personnel”

++ See table S1b for year by year definitions of “modern form of contraception”

Table S1b

Definition of Terms

| Survey Year | “Skilled health personnel” for ANC | Other Options | |
| --- | --- | --- | --- |
| 2000 | Health professional | Trained traditional birth attendant, untrained traditional birth attendant, other, no one | |
| 2005 | Health professional | Trained traditional birth attendant, untrained traditional birth attendant, community health agent, other, no one | |
| 2011 2014 | Doctor, nurse, midwife | Health extension worker (HEW), other health personnel, trained traditional birth attendant, untrained traditional birth attendant, voluntary community health worker (VCHW), other, no one | |
|  | “Skilled health personnel” for birth | Other options | |
| 2000 | Health professional | Trained traditional birth attendant, untrained traditional birth attendant, relative / friend, neighbor, other, no one | |
| 2005 | Health professional | Trained traditional birth attendant, untrained traditional birth attendant, community health agent, relative / friend, other, no one | |
| 2011 2014 | Doctor, nurse, midwife | HEW, other health personnel, trained traditional birth attendant, untrained traditional birth attendant, VCHW, relative / friend, other | |
|  | “Modern” form of contraception | | Other options |
| 2000 | Female sterilization, male sterilization, pill, intrauterine device (IUD), injections, implants, condom, diaphragm / foam / jelly | | Rhythm or periodic abstinence, withdrawal |
| 2005 | Female sterilization, male sterilization, pill, IUD, injections, implants, condom, diaphragm / foam / jelly, lactational amenorrhea method | | Standard days method, rhythm method, withdrawal |
| 2011 2014 | Female sterilization, male sterilization, pill, IUD, injectables, implants, male condom, female condom, lactational amenorrhea method | | Standard days method, rhythm method, withdrawal, emergency contraception |

Table S2

Test of Dominance between Concentration Curves

|  | Data 1  Survey Year | Data 2 Survey Year | Rule | |
| --- | --- | --- | --- | --- |
|  |  |  | mca | iup |
| Stunting | 2000 | 2014 | Non-dominance | Non-dominance |
| Wasting | 2000 | 2014 | 2014 dominates 2000 | Non-dominance |
| Underweight | 2000 | 2014 | 2014 dominates 2000 | Non-dominance |
| Measles vaccination | 2000 | 2011 | 2014 dominates 2000 | Curves cross |
| Full immunization | 2000 | 2011 | Non-dominance | Curves cross |
| Contraception | 2000 | 2014 | 2014 dominates 2000 | Non-dominance |
| ANC4+ | 2000 | 2014 | 2014 dominates 2000 | Non-dominance |
| SBA | 2000 | 2014 | 2014 dominates 2000 | 2014 dominates 2000 |

*Notes*: Test of dominance for concentration curves is based on O’Donnell et al. [15]. Significance level is 5% and the number of evenly spaced quintiles points is 19 (from 5% to 95%). The test is not applicable to the mortality concentration curves we developed in this study. However, curves cross in all illustrations of mortality concentration curves. The dominance test rules, mca and iup, respectively denote the multiple comparison approach and the intersection union principle.
